# Fabrication of Cellulose Nanocrystal/Chitosan Hydrogel for Controlled Drug Release

**DOI:** 10.3390/nano9020253

**Published:** 2019-02-13

**Authors:** Qinghua Xu, Yunzhong Ji, Qiucun Sun, Yingjuan Fu, Yongjian Xu, Liqiang Jin

**Affiliations:** 1 State Key Laboratory of Biobased Material and Green Papermaking, Qilu University of Technology (Shandong Academy of Sciences), Jinan 250353, China; jyzqilu@163.com (Y.J.); qiucunsun@163.com (Q.S.); fyj@qlu.edu.cn (Y.F.); 2 Shaanxi Province Key Lab of Paper Technology and Specialty Paper, Shaanxi University of Science and Technology, Xi’an 710021, China; xuyongjian@sust.edu.cn; 3 College of Leather Chemical and Engineering, Qilu University of Technology (Shandong Academy of Sciences), Jinan 250353, China

**Keywords:** cellulose nanocrystal, periodate oxidation, chitosan, hydrogel, drug delivery

## Abstract

In this work, a novel nanocomposite hydrogel based on cellulose nanocrystal (CNC) and chitosan (CS) was fabricated and applied as a carrier for the controlled delivery of theophylline. CNC was firstly periodate-oxidized to obtain dialdehyde nanocellulose (DACNC). Then, chitosan was crosslinked using DACNC as both the matrix and crosslinker in different weight ratios, to fabricate CNC/CS composites. The prepared composites were characterized using Fourier transform infrared spectroscopy (FT-IR), X-ray diffraction pattern (XRD), scanning electron microscopy (SEM), zeta potential measurement and swelling ratio tests. FT-IR results confirmed the successful reaction between the free amino groups on chitosan and the aldehyde groups on DACNC. With the increase of chitosan percentage in the hydrogel, the isoelectric point was shifted towards an alkaline pH, which was probably caused by the higher content of free amino groups. The swelling ratio of the composite also increased, which may have been due to the decrease of crosslinking density. Because the swelling ratio of the drug-loaded hydrogels differed under varied pH values, the cumulative drug release percentage of the composite hydrogel was achieved to approximately 85% and 23% in the gastric (pH 1.5) and intestinal (pH 7.4) fluids, respectively. Therefore, CNC/CS hydrogel has application potential as a theophylline carrier.

## 1. Introduction

Environmental intelligent hydrogels have attracted extensive interest in biomedical fields, such as tissue engineering, biosensors, and drug delivery [1,2]. To formulate drug delivery systems with desirable mechanical properties and drug releasing profiles, polymers with a broad variety of physicochemical properties are needed.

Chitosan is the second most abundant biomaterial on the earth, which is obtained by N-deacetylation of chitin [3]. It is nontoxic, biocompatible, biodegradable, and antibacterial in nature. These properties have led to significant research towards biomedical and pharmaceutical applications [4,5,6,7], such as drug delivery, tissue engineering, wound care dressing etc. However, the poor mechanical strength and poor stability in acidic aqueous solution greatly limits the applications of chitosan [8].

Nanocellulose can also be used as a promising material for the controlled release of drugs due to its unique properties such as high water-holding capacity, high crystallinity, large surface area, and biocompatibility [9,10]. Kolakovic et al. [11] reported the application of nanofibrillar cellulose as a matrix-former material for long-lasting (up to three months) sustained drug delivery. The nanocellulose aerogel scaffolds made from red pepper (RC) and microcrystalline cellulose (MCC) release the drug immediately, while bacterial cellulose (BC), quince seed (QC), and TEMPO-oxidized birch cellulose-based (TC) aerogels show sustained drug release [12]. Shao et al. [13] prepared an antibiotic drug tetracycline hydrochloride (TCH)-loaded BC composite membranes, and evaluated the drug release, antibacterial activity, and biocompatibility. The results showed that the incorporation of BC matrix to load TCH was able to control the release.

Recently, cellulose and chitosan as composite components have attracted much attention due to their strong chemical similarities and biocompatibility of the polysaccharide structures [14]. Nanocellulose could be used as reinforcement in nanocomposites due to their high mechanical properties, large surface area, and aspect ratio. Its incorporation in chitosan could also improve the mechanical properties and stability of chitosan-based composites. Sarkar et al. [15] developed a CNF/chitosan transdermal film for the delivery of ketorolac tromethamine where the CNFs acted as an elegant nanometric carrier. The release profile indicated that the drug release rate was sustained with the incorporation of CNFs into chitosan. Biocompatible chitosan/poly (vinyl pyrrolidone)/nanocellulose (CPN) composites were prepared by a casting method [16]. The biological study suggested that the composite may be promising as a wound-healing material for biomedical application. A drug delivery system based on oxidized cellulose nanocrystal (CNC) with chitosan oligosaccharide was developed [17]. Primary hydroxyl groups on the surface of CNC were selectively oxidized to carboxylic acid groups using TEMPO-mediated oxidation. The amino groups of chitosan oligosaccharide were then reacted with carboxylic acid groups on oxidized CNC via carbodiimide. The composite had potential applications as a fast-response drug carrier in wound dressings and local drug delivery to the oral cavity, such as in treatment of periodontal cavities. Nanocellulose-reinforced chitosan hydrogel was prepared using glutaraldehyde as the crosslinker for the oral administration of curcumin [18]. It can be suggested as a promising candidate for stomach-specific drug delivery.

The preparation technique of chitosan–cellulose nanowhisker composites affects the distribution of nanocellulose in chitosan and hence influence the interaction between them. The enhancement of the properties of the final nanocomposite is highly dependent on the level of dispersion and the final morphology of the nanocomposites [19]. Poor dispersion of nanofillers in polymeric matrices leads to composites with inadequate properties. Previously reported results stated that the distribution of concentrated cellulose suspension in chitosan solution was problematic and resulted in poor properties [20]. Different processes have been employed for the production of CNC-chitosan composites [14]. Generally, the intermolecular interactions between CNC and chitosan are based on H-bond, van der Waals forces, and ionic and/or covalent bonds, depending on the processing route [21]. Compared to H-bond, van der Waals forces, and ionic bonds, covalent bonds can provide a much stronger linkage between CNC and chitosan, thus obtaining composites with high stability and mechanical properties. Karim et al. [22] prepared a membrane with cellulose nanocrystals as a functional entity in chitosan through freeze-drying, followed by a compacting process. Chitosan was bound to cellulose nanowhiskers (CNW) in a nanoporous membrane structure and crosslinked with gluteraldehyde vapors for further stabilization. The membrane successfully removed 98% of Victoria Blue 2B, 84% of Methyl Violet 2B, and 70% of Rhodamine 6 G dyes after 24 h. De Mesquita et al. [23] prepared chitosan-CNW multilayers using a layer-by-layer assembly of highly deacetylated chitosan (cationic phase) and CNW (anionic phase). H-bonds and electrostatic interactions between amino groups on chitosan and sulphate groups on CNW resulted in the growth of multilayered films. In another work [24], they prepared bio-based nanocomposites through covalent linkage between chitosan and cellulose nanocrystals (CNCs), functionalized with methyl adipoyl chloride. However, surface modification of CNCs was performed in toluene, resulting in a very complex separation process due to its toxicity. Karim et al. [14] prepared chitosan–CNW nanocomposite membranes by solution mixing and then a freeze-drying process, with their ratios ranged from 1:1 to 1:3. Sanchez–Salvador [25] prepared a nanocellulose-graft–chitosan composite. Recycled cellulose pulp with a TEMPO-mediated oxidation pretreatment was used as the raw material to produce CNFs. The amino group on chitosan attacks the carboxylic group on oxidized cellulose to obtain the nanocellulose-graft–chitosan. Harkins et al. [26] fabricated a chitosan–cellulose biocomposite by dissolution of chitosan and cellulose, using a green solvent butyl methylimmidazolium chloride (BMIm^+^Cl^−^). The composites were found to inhibit the growth of both Gram-positive and Gram-negative microorganisms. Genipin-crosslinked complex microspheres made of the combination of ethyl cellulose and chitosan, were prepared by a spray-drying method [27]. Rifabutin (RBT), an anti-tuberculosis drug was loaded into the composite as the model drug to investigate the release behavior. It was found that the complex microspheres possessed biphasic release and long-time pulmonary retention features.

In this work, cellulose nanocrystal (CNC) was firstly periodate-oxidized to obtain dialdehyde nanocellulose (DACNC). Then, chitosan (CS) was crosslinked using DACNC as both the matrix and crosslinker in different weight ratios, to fabricate CNC/CS composites. The aldehyde groups on DACNC reacted with the free amino groups on CS through a Schiff base reaction. Thus, a strong covalent linkage between nanocellulose and chitosan was formed without using any toxic chemical crosslinker, such as the commonly used glutaraldehyde. The covalent linkages between CNC and chitosan could allow a uniform distribution and the incorporation of high amounts of nanocellulose in the composite. An in vitro drug release performance of the obtained CNC/CS composites was investigated using theophylline as the model drug.

## 2. Materials and Methods

### 2.1. Materials

Cellulose nanocrystal was provided by Tianjin Woodelf Biotechnology Co. Ltd., China. Sodium periodate and chitosan with a low molecular weight and a deacetylation degree of 90% were purchased from Sigma–Aldrich Co. Ltd., Beijing, China.

### 2.2. Preparation of DACNC and CNC/CS Hydrogel

CNC was periodate-oxidized to obtain DACNC according to the method by Jin et al. [28], and 9 mmol sodium periodate per gram of cellulose nanocrystal was used. Chitosan was dissolved in 2% acetic acid aqueous solution to get a chitosan solution (3% *w*/*v*). The dialdehyde cellulose nanocrystals were mixed with chitosan at the mass ratio of 1:1, 1:2, and 1:5, respectively, for a reaction of 8 h at 45 °C. Unreacted chitosan and DACNC were removed by washing with deionized water several times. The obtained composite hydrogels, namely CNC/CS1, CNC/CS2, and CNC/CS3, were freeze-dried for the subsequent characterization.

### 2.3. Characterization

#### 2.3.1. Elemental Analysis

The percentage of carbon, hydrogen, nitrogen, and sulfur of DACNC, chitosan, and the hydrogels were determined with a Vario EL Ш Elemental Analyzer (Elementar, Frankfurt, Germany). The quantity of each element was expressed in a percentage of dry mass.

#### 2.3.2. Fourier Transform Infrared Spectroscopy 

FT-IR spectra of CNC, CS, and CNC/CS composites were conducted on an IRPrestige-21 Fourier Transform Infrared Spectrometer (Shimadzu Company, Kyoto, Japan). The spectra were recorded with width ranging from 400 to 4000 cm^−1^, and a resolution of 2 cm^−1^.

#### 2.3.3. X-ray Diffraction Analysis

X-ray diffraction analyses were performed with a D8 Powder X-ray Diffractometer (Bruker AXS, Karlsruhe, Germany), which was equipped with a CuXa X-ray tube.

#### 2.3.4. Scanning Electron Microscopy 

Swollen hydrogels were freeze-dried and observed with a Hitachi Regulus 8220 field emission scanning electron microscopy (Tokyo, Japan) equipped with an energy-dispersive X-ray spectrometer. Before observation, the samples were sputter-coated with gold for 100 s.

#### 2.3.5. Zeta Potential Measurement

The fresh hydrogel samples were dispersed in deionized water. The pH of the system was then adjusted using HCl or NaOH solution. The samples with varied pH were sonicated for approximately 10 min, and the zeta potential of composite hydrogel samples at varied pH was determined using a Malvern Zetasizer Nano ZS90 (Malvern, UK). The measurement temperature was set at 25.0 ± 0.1 °C and the data was processed by Malvern Zetasizer Software v7.11.

#### 2.3.6. Swelling Ratio

The swelling ratios of the hydrogels were measured at room temperature with varied pH from 3 to 10, according to the method of Kang et al. [29]. Of each hydrogel, 0.2 g was put into 20 mL of buffer solution and gently stirred for 8 h to get the equilibrium swelling. The hydrogel was then retrieved by centrifugation. The swollen hydrogels were removed from the solutions and then weighed as soon as possible. The swelling ratios were determined according to the following Equation (1):

(1) $$\mathrm{Swelling}\;\mathrm{ratio}\;\left(\%\right)=\frac{\mathrm{wetweight}-\mathrm{dryweight}}{\mathrm{dryweight}}\times 100$$

### 2.4. Drug Loading

Theophylline was used as the model drug for drug loading and release. The dried hydrogels were immersed in 1 mg/mL aqueous theophylline solution for 24 h in darkness. Drug contents in the hydrogel were calculated according to the original concentration of theophylline and the concentration of theophylline in the solution after loading, which could be quantified using an Agilent 8453 UV-Vis spectrophotometer at a wavelength of 273 nm. The drug encapsulation efficiency was calculated using Equation (2):

(2) $$\mathrm{Encapsulation}\;\mathrm{efficiency}\;\left(\%\right)=\frac{\mathrm{Amount}\;\mathrm{of}\;\mathrm{theophylline}\;\mathrm{in}\;\mathrm{the}\;\mathrm{hydrogel}}{\mathrm{Amount}\;\mathrm{of}\;\mathrm{the}\;\mathrm{ophylline}\;\mathrm{in}\;\mathrm{the}\;\mathrm{initial}\;\mathrm{solution}}\times 100$$

### 2.5. In Vitro Drug Release

Drug release experiments were performed in a shaker at a speed of 50 rpm at 37 °C, at pH 1.5 and 7.4, respectively. The drug-loaded hydrogels were placed in a buffer solution and 5 mL of solution was collected at 1 h intervals for the determination of drug content using a Perkin Elmer lambda 25 UV-Vis spectrophotometer at 356 nm. An equal volume of the same solution medium was added back to maintain a constant volume. All samples were conducted in triplicate in vitro release tests. The concentration of the drug in the different buffer solutions was calculated by the calibrated standard curves.

## 3. Results and Discussion

### 3.1. Preparation and Characterization of CNC/CS Nanocomposite Hydrogel

**Scheme 1:**
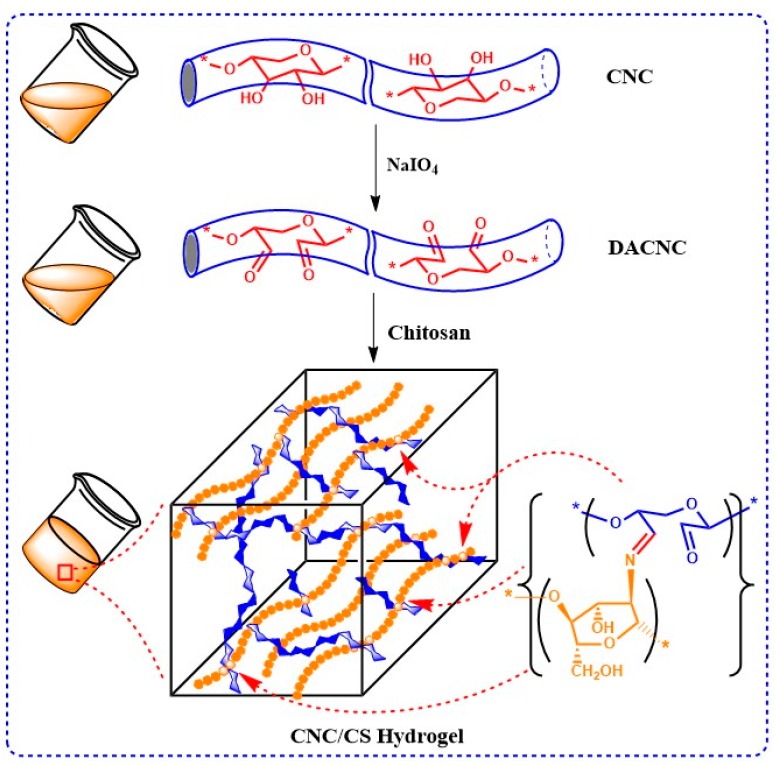
Schematic representation of reaction between dialdehyde cellulose nanocrystal (DACNC) and chitosan (CS).

The schematic pathway for sodium periodate oxidation of CNC and fabrication of the nanocellulose–chitosan composite is shown in Scheme 1. Cellulose nanocrystal (CNC), prepared from fully bleached hardwood kraft pulp by sulfuric acid hydrolysis, was oxidized by sodium periodate to yield dialdehyde cellulose nanocrystal (DACNC) with an aldehyde group content of 8.81 mmol/g. DACNC was then reacted with CS to obtain a cellulose nanocrystal/chitosan (CNC/CS) composite hydrogel through a Schiff base reaction between aldehyde groups on DACNC and free amino groups on chitosan.

Three hydrogels were prepared by varying the weight ratio of DACNC and CS. Table 1 lists the elemental composition of CS, DACNC, and the hydrogel. DACNC did not have any nitrogen, and the presence of nitrogen in the hydrogel indicated the incorporation of chitosan. With the increase of chitosan ratio, the nitrogen content increased in the hydrogel, indicating a higher incorporation percentage of chitosan in the composite. The oxygen content in the hydrogel samples decreased with the increase of chitosan ratio. This may have resulted from the reaction between amino and dialdehyde groups, during which oxygen was turned into water. Small amounts of sulfur (0.1%−0.2%) were detected in DACNC and the hydrogel samples, resulting from the sulfate ester groups during the sulfuric acid hydrolysis of the bleached kraft pulp. The amount of sulfate ester groups on the surface of nanowhiskers was very small, which may have had a minimal effect on surface reactions [30]. Since the nitrogen in the hydrogel all originated from chitosan, the weight percentage of DACNC and chitosan in the hydrogel could be determined by comparing the nitrogen content of the hydrogel with that of chitosan. The results are listed in Table 2. For CNC/CS1 and CNC/CS2, the nanocellulose and chitosan weight percentage was 1:1 and 1:2, respectively, almost the same as the ratio during their preparation. Meanwhile, the weight percentage of nanocellulose and chitosan for CNC/CS3 was 1:4, lower than its original preparation ratio (1:5), suggesting that the DACNC could crosslink as much as four times its own weight of chitosan.

**Figure 1:**
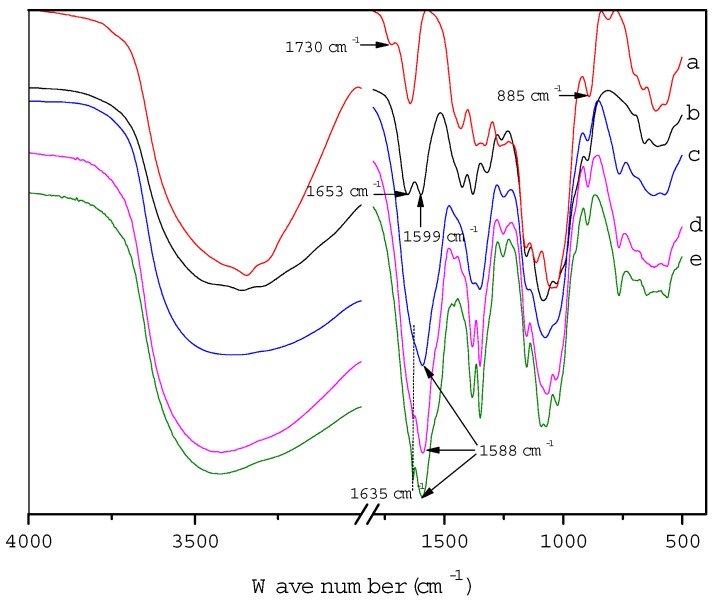
FT-IR spectra of (**a**) DACNC; (**b**) CS; (**c**) CNC/CS3; (**d**) CNC/CS2; (**e**) CNC/CS1.

Figure 1 shows the FT-IR spectra of DACNC, CS and the CNC/CS hydrogels. In the spectra of DACNC (Figure 1a), a band at 1730 cm^−1^ was observed, corresponding to the C=O stretching vibration of the dialdehyde cellulose nanocrystal. An apparent absorption peak at 883 cm^−1^ caused by the formation of hemiacetal bonds between newly achieved aldehyde groups and their neighboring hydroxyl groups was also observed. The results indicated a successful oxidization of the hydroxyl groups on the molecular chain of CNC to aldehyde groups. In the spectra of CS (Figure 1b), the broad peak at 3408 cm^−1^ corresponded to the O–H and N–H stretching vibration. The peaks at 1653 and 1599 cm^−1^ represented the –C=O bending vibration in the amide I band and N–H bending vibration, respectively. FT-IR spectra of the nanocomposite hydrogels were very similar (Figure 1c–e). The absorption band at 1730 cm^−1^ disappeared while a new adsorption band at 1640 cm^−1^ appeared, which corresponded to the stretching vibration of –C=N group. These results indicated the successful reaction between DACNC and CS.

**Figure 2:**
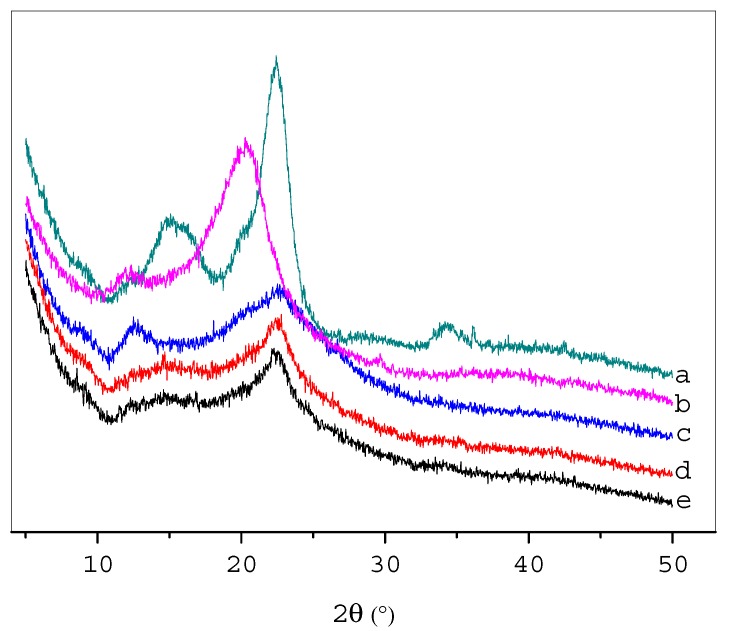
X-ray diffraction patterns of (**a**) CNC; (**b**) CS; **c**, CNC/CS3; (**d**) CNC/CS2; and (**e**) CNC/CS1.

The crystal structure of CNC, CS, and the CNC/CS hydrogel were characterized by XRD and are shown in Figure 2. The X-ray diffractograms of CNC (Figure 2a) show diffraction peaks at 2θ = 14°–18°, 22.5°, and 34.5°, which corresponded to (1-10), (110), (200) and (004) crystal planes of cellulose, indicating a crystal structure of cellulose I. The XRD profile of the chitosan (Figure 2b) exhibited the characteristic peaks at 2θ of 12.5° and 20.1°, due to the crystalline nature of chitosan [15]. All the hydrogels showed diffraction peaks at 2θ of 22.5°, while the intensity of peaks at 2θ of 14°–16° and 34.5° decreased. For the CNC/CS3 (Figure 2c), these two peaks disappeared, indicating that the cellulose crystallinity was almost lost. Meanwhile, a characteristic peak at 2θ of 12.5° for chitosan was observed, due to the higher chitosan percentage in the composite. A similar observation was reported previously by Li et al. [31], that increase in CNF percentage in the composites led to an increase in the crystalline nature of the composites.

**Figure 3:**
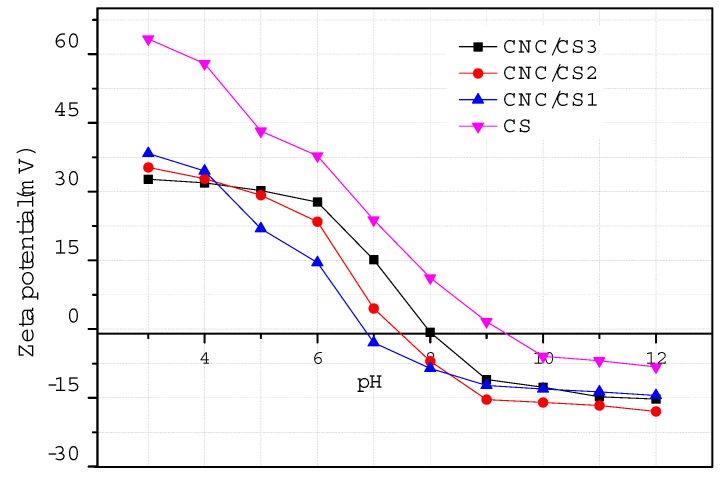
Zeta potential of CNC/CS composites.

Zeta potential of CS and CNC/CS composites at varied pH was demonstrated in Figure 3. The zeta potential was positive at low pH and negative at high pH for all samples, suggesting their amphoteric nature. It was found that the isoelectric point of CNC/CS1, CNC/CS2, and CNC/CS3 were 6.9, 7.5, and 8.0, respectively. The composite hydrogel possessed cationic (amino) and anionic (sulfate ester groups, –O–SO_3_^−^) ionizable surface groups. Its zeta potential was positive at low pH due to the protonation of free amino groups. With the increase of the pH value, the zeta potential decreased and became negative, resulting from the deprotonation of primary amine groups and dissociation of sulfate groups on the surface. The isoelectric point for the composite hydrogel was in the order of CNC/CS1 < CNC/CS2 < CNC/CS3. With the increase of chitosan percentage in the composite, the isoelectric point was shifted towards alkaline pH, which was probably caused by the higher content of free amino groups [32].

**Figure 4:**
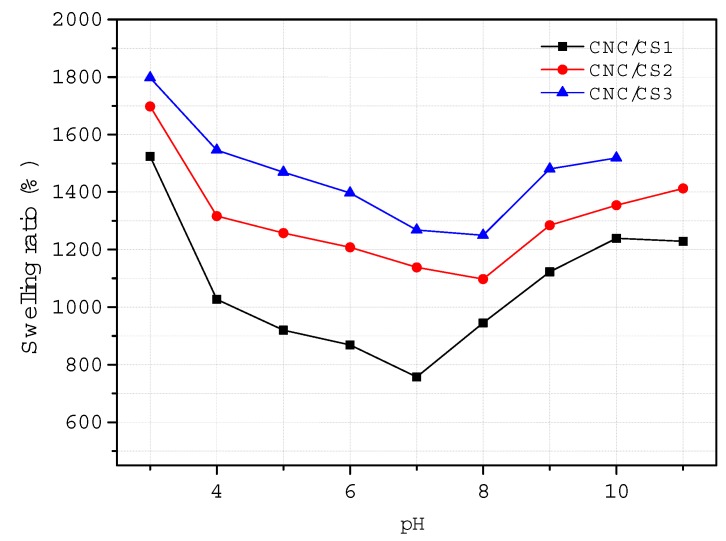
Swelling ratio of CNC/CS composite hydrogel at varied pH.

Swelling ratio is an important property for a hydrogel when considering its application in drug delivery. Figure 4 shows the swelling ratio of CNC/CS composite hydrogel at varied pH. It also exhibited a corresponding dependency on pH, as the zeta potential did. It is well known that the swelling of hydrogel is induced by the electrostatic repulsion of the ionic charges of its network [33]. At the lower pH, the high zeta potential would result in an electrostatic repulsion, inducing the swelling of the hydrogel. With the increase of the pH, the repulsion was mitigated due to the decrease of the surface charges, which would induce the deswelling of the hydrogel. Minimum swelling occurred at the isoelectric point. When the pH was higher than the isoelectric point, the swelling ratio increased due to the negative surface charge of the composites. With the increase of chitosan weight percentage in the composite, the swelling ratio increased. CNC/CS3 shows the highest swelling ratio, while CNC/CS1 shows the lowest. Higher CNC percentage in the composite resulted in higher crosslinking density and thus the formation of a more rigid hydrogel structure, which led to a decrease in swelling ratio. Furthermore, the presence of CNC in the hydrogel provided barrier properties that prohibited water molecules from getting into the composite [20,34]. The results were in agreement with those reported by Sarkar et al. [15]. The researchers fabricated cellulose nanofibrils/chitosan films by mixing them together for drug-controlled release, and also found a decrease in the swelling of the films with an increase in the CNF concentration.

**Figure 5:**
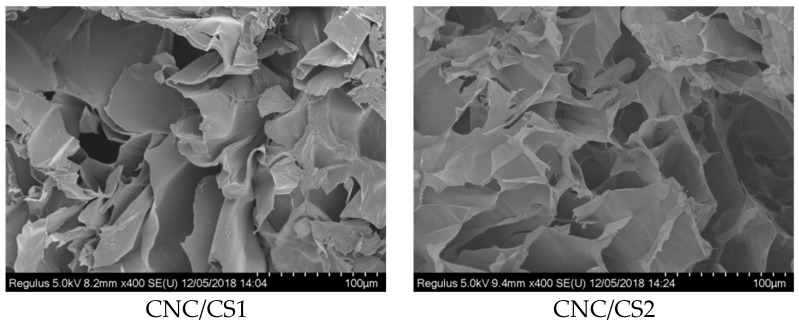

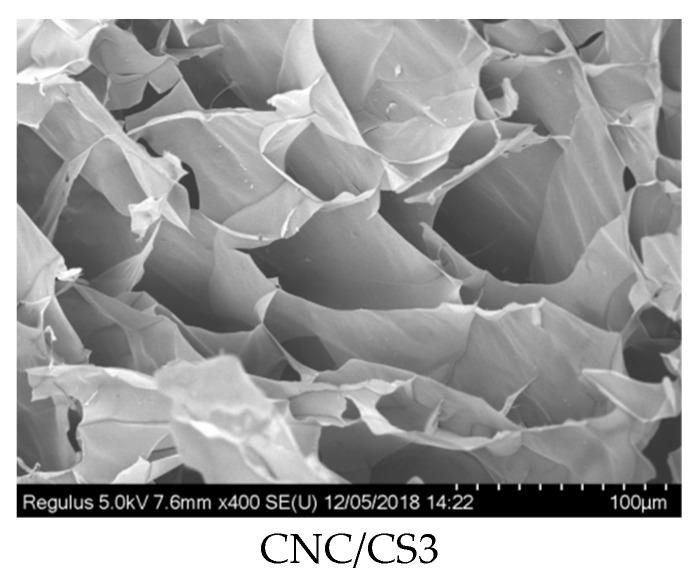
FE-SEM images of CNC/CS1, CNC/CS2, and CNC/CS3.

The hydrogels were freeze-dried to form aerogels, which were highly porous and lightweight, with a density of 0.0188, 0.0191, and 0.0205 g/cm^3^ for CNC/CS1, CNC/CS2, and CNC/CS3, respectively. The ultra-light weight of the composites may be due to the incorporation of high amounts of CNC and high porosity. The morphology of freeze-dried CNC/CS1, CNC/CS2, and CNC/CS3 were observed by FE-SEM, as demonstrated in Figure 5. Naturally, all of the hydrogels exhibited porous and interconnected microstructures. The pore size increased as the chitosan percentage increased. The gradual increase in pore size of the hydrogels could have been due to the decreased degree of crosslinking, and because more water was taken into the hydrogel after swelling. This corresponded well with the results of the swelling ratio. Similar findings were reported by Dash et al. [35], who found that the crosslinking between gelatin and cellulose nanowhiskers offered more intermolecular association, which in turn affected the pore structure, size, and their distribution. When the contents of CNC increased to 50% (CNC/CS1), the pore structure was damaged by fractures, which may have been due to the aggregation of excessive amounts of CNC in the composite. CNC/CS2 and CNC/CS3, with CNC contents of 33% and 25%, respectively, both displayed a homogeneous structure, indicating a high level of dispersion of cellulose nanocrystal in the composites. Bao et al. [36] fabricated CNW-reinforced chitosan–xylan nanocomposite films by solution mixing and found that the pore structure was damaged with fractures appearing when the contents of CNW were further improved to 16%. Li et al. [31] prepared composite films of chitosan reinforced with cellulose nanowhiskers by mixing solutions of these two components. A homogeneous structure for the composite films could be obtained with a good dispersion of CNW up to an additional amount of 20%. The preparation process in this work could be the reason for a higher amount of CNC incorporation than those reported. Generally, the pore size, structure, and the porosity of the composites could be tailored by varying the ratio between DACNC and chitosan, and thus influence their application performance.

### 3.2. Drug Loading and In Vitro Drug Release Study

Theophylline was used as a drug model to investigate the performance of the CNC/CS composite in drug release. Theophylline is a xanthine derivative, which is mainly used in the chronic treatment of bronchial asthma and bronchospastic diseases [37]. The encapsulation efficiency of theophylline for the hydrogels is listed in Table 3. CNC/CS3 shows the maximum encapsulation efficiency, while CNC/CS1 shows the minimum, which may have resulted from the increasing swelling ratio as the chitosan percentage in the composite increased.

**Figure 6:**
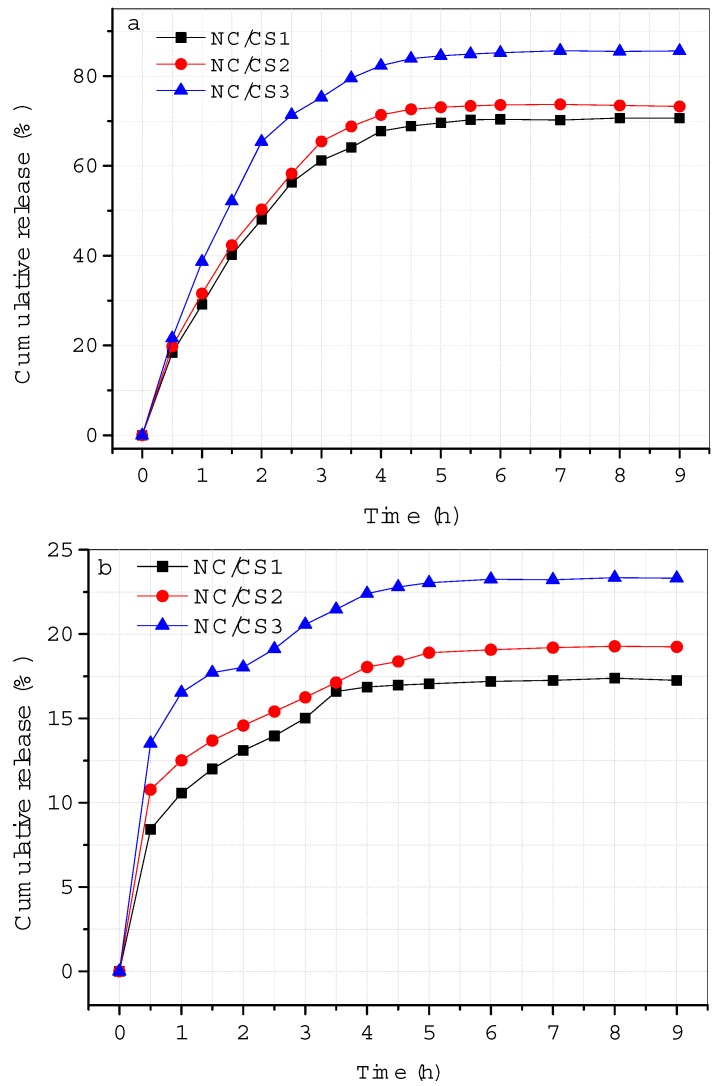
In vitro cumulative drug release from the drug-loaded hydrogels in (**a**) pH 1.5 and (**b**) pH 7.4 buffer solutions at 37 °C.

The release profiles in pH 1.5 and 7.4 buffer solutions are demonstrated in Figure 6. The drug release profiles of the CNC/CS composite showed burst releases of the drug from 1 to 3 h, and then a gradual plateauing of release was observed. The release rate increased as the chitosan percentage in the composites increased, due to the increase of the pore size and porosity. The cumulative drug release at pH 1.5 was significantly greater than that achieved at pH 7.4, resulting from the higher swelling ratio of the hydrogels in the acid environment. At the same pH, NCC/CS3 gave the highest cumulative release, approximately 85% at pH 1.5 and 23% at pH 7.4 after continuous release of 5 h. CNC/CS hydrogels demonstrated excellent drug-controlled release behaviors and can therefore be used as potential carriers for gastric-specific drug delivery.

## 4. Conclusions

CNC/CS composites were fabricated using DACNC and chitosan at varied weight ratios. FT-IR results evidenced the successful reaction between aldehyde groups on DACNC and amino groups on chitosan. The isoelectric point and swelling ratio increased with the increase of the chitosan percentage in the composites. An in vitro drug release study was performed using theophylline as the model drug. The cumulative drug release at pH 1.5 was significantly greater than that achieved at pH 7.4. CNC/CS3 gave the highest cumulative release, approximately 85% at pH 1.5 and 23% at pH 7.4. CNC/CS composites demonstrated excellent drug-controlled release behaviors, and can be used as potential carriers for gastric-specific drug delivery. New composites based on DACNC and some other biocompatible natural polymers, such as gelatin, and their performance in controlled drug release could be the subject of future research. The focus will be on the tailoring of porosity and drug-releasing rates of the composites by varying the oxidation degree of DACNC and its loading in the composites.

## Figures and Tables

**Table 1:** Elemental analysis of cellulose nanocrystal/chitosan (CNC/CS) hydrogels.

Sample	Elemental Composition (%)				
	C	O ^a^	N	H	S
CS	38.980	47.347	6.773	6.900	
DACNC	37.66	56.28		5.87	0.190
CNC/CS1	38.804	51.310	3.318	5.688	0.184
CNC/CS2	38.420	50.295	4.536	6.577	0.172
CNC/CS3	37.551	49.773	5.406	7.143	0.127

^a^ O% = 100% − (%C + %H + %S + %N).

**Table 2:** Cellulose nanocrystal and chitosan weight percentage in the composites.

Composites	CNC (%)	CS (%)	CNC:CS
CNC/CS1	51.0	49.0	1:1
CNC/CS2	33.0	67.0	1:2
CNC/CS3	20.2	79.8	1:4

**Table 3:** Encapsulation efficiency of theophylline for different hydrogels.

Samples	CNC/CS1	CNC/CS2	CNC/CS3
Encapsulation efficiency (%)	85.16	89.27	92.31
